# Managing Deviating EQA Results: A Survey to Assess the Corrective and Preventive Actions of Medical Laboratories Testing for Oncological Biomarkers

**DOI:** 10.3390/diagnostics10100837

**Published:** 2020-10-18

**Authors:** Cleo Keppens, Ed Schuuring, Elisabeth MC Dequeker

**Affiliations:** 1 Department of Public Health and Primary Care, Biomedical Quality Assurance Research Unit, University of Leuven, Kapucijnenvoer 35d, 3000 Leuven, Belgium; cleo.keppens@kuleuven.be; 2 Department of Pathology, University Medical Center Groningen (UMCG), University of Groningen, Hanzeplein 1, 9700 RB Groningen, The Netherlands; e.schuuring@umcg.nl

**Keywords:** external quality assessment, corrective action, preventive action, quality management, ISO 15189, colorectal cancer, non-small cell lung cancer, laboratory accreditation

## Abstract

Laboratories testing predictive biomarkers in lung and colorectal cancer are advised to participate in external quality assessment (EQA) schemes. This study aimed to investigate which corrective actions were taken by laboratories if predetermined performance criteria were not met, to ultimately improve current test practices. EQA participants from the European Society of Pathology between 2014 and 2018 for lung and colorectal cancer were contacted, if they had at least one analysis error or test failure in the provided cases, to complete a survey. For 72.4% of 514 deviating EQA results, an appropriate action was performed, most often including staff training (15.2%) and protocol revisions (14.6%). Main assigned persons were the molecular biologist (40.0%) and pathologist (46.5%). A change in test method or the use of complex techniques, such as next-generation sequencing, required more training and the involvement of dedicated personnel to reduce future test failures. The majority of participants adhered to ISO 15189 and implemented suitable actions by designated staff, not limited to accredited laboratories. However, for 27.6% of cases (by 20 laboratories) no corrective action was taken, especially for pre-analytic problems and complex techniques. The surveys were feasible to request information on results follow-up and further recommendations were provided.

## 1. Introduction

In non-small cell lung cancer (NSCLC) and metastatic colorectal cancer (mCRC), testing of tumor-specific predictive biomarkers is routine practice in medical laboratories [1].

It is the responsibility of the laboratories performing the molecular test to take measures towards improvement. Diagnostic laboratories are thus recommended to proceed towards accreditation according to the International Organization for Standardization (ISO) standard 15189:2012 or national equivalents like CAP 15189 [1,2,3]. Accredited laboratories should fulfill the requirements related to management of procedures such as the provision of adequate facilities, trained staff and documentation. This also entails that diagnostic laboratories should participate in an interlaboratory comparison program such as external quality assessment (EQA) schemes [3]. These programs should preferably be accredited according to ISO 17043:2010 [4], mimic patient samples as closely as possible, and check the entire examination process [3]. Laboratories should document and monitor their EQA scheme results. If predetermined performance criteria are not met, laboratories should take corrective and preventive actions (CAPAs): corrective actions to eliminate the cause(s) of existing nonconformities and preventive actions to eliminate the occurrence of potential deviations.

Several national and international EQA schemes are available for testing common predictive biomarkers in NSCLC and mCRC, such as those by the European Society of Pathology (ESP) [5], the European Molecular Genetics Quality Network in Europe [6], the College of American Pathologists (CAP) in the USA [7], NordiQC [8], the Royal College of Pathologists of Australasia Quality Assurance Programs for Australasia [9], and national schemes organized in Canada, Germany, France, Italy, Spain, the United Kingdom, the Netherlands, and Belgium [10,11,12,13,14,15,16,17].

In 2016, a flowchart was proposed by Kristensen and Meijer to guide laboratories in handling a deviating EQA result in quantitative laboratory medicine, indicating the responsibilities of both the participants and EQA providers or manufacturers, depending on the deviation type [18]. In 2018, the group of Sciacovelli et al. reported a set of critical aspects that EQA providers and laboratory professionals should control in order to guarantee effective EQA management and compliance with ISO 15189, in a checklist used to identify causes of unsatisfactory EQA performance [19]. In that same year, longitudinal data from the ESP EQA schemes revealed that laboratory accreditation, increased experience, and a research setting improved EQA performance when implementing novel biomarkers [20].

Biomarker analyses include predictive markers which are required for appropriate targeted treatment decision-making or prognostic markers to obtain information on the patient’s overall cancer outcome [1,21,22]. Diagnostic laboratories are thus challenged to provide accurate and reproducible test results within an acceptable timeframe. There is currently no universal process for the response to poor performance by the EQA provider [23,24]. EQA providers could report persistent poor performers to governmental bodies, like the National Quality Assessment Advisory Panel (NQAAP) for GenQA, UK NEQAS, and EMQN [6,15,25], or rely on national accreditation bodies in the absence of such bodies [24]. EQA providers can however support the participants in improving their service via, for instance, the provision of reference material, methodological advice, or support in quality management through reviewing CAPA plans [23,24]. Indeed, some EQA providers such as CAP or UKNEQAS request a corrective action upon poor performance for review [7,15].

Although the EQA schemes detect and report on participants’ overall performance, it is currently unknown how laboratories process the EQA feedback they received or how they handle deviating EQA results.

In this paper we analyzed which actions were taken by participants in the ESP Lung and Colon EQA schemes [5] between 2014 and 2018 in case of deviating EQA results, by sending additional research surveys besides the data collected during the schemes. We also evaluated whether the (extent and type of) actions differed for various laboratory characteristics and which ones were more likely to enhance performance in future EQA schemes. The analyses aim to raise awareness on contemporary error management strategies in laboratories testing these biomarkers, to ultimately improve current test practices.

## 2. Materials and Methods

The ESP schemes were organized according to the ISO 17043 standard [4] for proficiency testing and the guideline on the requirements of EQA programs in molecular pathology [23]. Details on sample selection and preparation, validation by the reference laboratories, and distribution were previously described [26,27]. Laboratories could opt to participate to several offered subschemes for testing of common predictive markers in NSCLC and mCRC (Table 1). Laboratories were requested to analyze all samples using their routine test methodology within 14 calendar days and to return an electronic datasheet on the cases’ outcomes (positive or negative for fluorescence in-situ hybridization (FISH)/immunohistochemistry (IHC) and the exact mutation/wild-type (WT) status for variant analysis) and additional information on their settings. Scheme results were assessed by a team of international experts as previously described [26,27]. Thereafter, results were released to the laboratories in the form of a general scheme report, participation certificate, and individual comments.

Between December 2015 and February 2019, laboratories with at least one incorrect outcome or test failure (i.e., no outcome available due to a technical analysis failure) for a given case were invited via e-mail to complete a survey, as described elsewhere [28]. Survey questions concerned (1) actions taken for the specific case(s) with deviating EQA results during that scheme and (2) general questions on how the laboratory usually manages unexpected or incorrect results in routine biomarker testing (Table S1). Root causes of deviating results were described previously [28]. This manuscript focuses on CAPAs to resolve deviating EQA results. For this type of work, no institutional review board (IRB) approval was needed at our institution.

Graphs were created using GraphPad Prism (version 8.3.0 of GraphPad Software, San Diego, CA, USA). Statistics were performed with SAS software (version 9.4 of the SAS System for Windows, SAS Institute Inc., Cary, NC, USA). To account for clustering of identical laboratories participating in different schemes (NSCLC vs. mCRC) and years, statistical generalized estimating equations (GEE) were applied. Binary outcome variables were analyzed using logistic regression models and ordinal and categorical outcome variables using proportional odds models. Results for both models are presented as odds ratios (ORs) with 95% confidence intervals (95% CI), in the format (*p*-value, OR [CI lower limit; CI upper limit]). Odds ratios higher/lower than 1 reflect a higher/lower probability for a higher level (+1 category for ordinal variables) or for the first level compared to the second level (for binary variables). For categorical variables, a global test was performed, and ORs from pairwise comparisons were calculated only if the global *p*-value was significant.

## 3. Results

Between 2015 and 2018, 2291 scheme participations were analyzed, during which 410 unique laboratories tested a total 21536 cases. Of those cases, 1167 were included in the survey because of a deviating result compared to the validated outcome. A total of 791 individual surveys were sent out, to 315 unique laboratories originating from 43 different countries. Of those, 325 (39.8%) responses were received from 185 unique laboratories (58.7%) in 34 countries. The average time to respond was 22.5 days (min. 1, max. 211, median 15 days) [28].

The 791 individual surveys included questions on 1167 cases that were incorrect or failed in the EQA schemes for NSCLC and mCRC. Information on how these cases were monitored further was received for 514 cases with a deviating EQA result (44.0%) (Table 1).

### 3.1. Management of Deviating EQA Scheme Results

The laboratories’ practices for managing the deviating EQA results observed in the 514 cases is shown in Table 2.

The majority of survey respondents reviewed the EQA results within the first week after release of the result by the EQA provider (58.5%, *n* = 407), and EQA results were always discussed with other team members in the laboratory (81.8%, *n* = 330). Most errors were noticed after result review (83.9%, *n* = 435), instead of before via a quality check in the laboratory.

For 72.4% cases, an appropriate CAPA was performed as response to the deviating EQA result. The most performed actions included staff training (15.2%) and protocol revisions (14.6%). For 56.0% of errors (*n* = 430), one person was designated to follow up the CAPA, while for the other 44.0% more than one person was assigned. The main assigned persons were the molecular biologist (40.0%) and pathologist (46.5%).

The type of corrective actions undertaken depended on the nature of the underlying error cause (Table S3), which were previously described in detail [28]. Staff trainings and protocol revisions were in most cases performed for issues related to the interpretation (60.3%, *n* = 78) or reagents (28.0%, *n* = 75), respectively. However, for 27.6% of the deviating EQA results, no CAPA was taken. In the cases where no action was performed (*n* = 142), underlying causes were reported to be due to problems with the received sample material (26.8%) and also interpretation or methodological problems (both 23.9%).

The greater majority (90.2%) of respondents indicated the importance of EQA participation to verify their performance on a scale of 10 points to be 7 or higher (Table 2). The majority of laboratories (84.1%, *n* = 315) indicated that the samples for the EQA scheme were not treated any differently compared to routine cases but the staff members manipulating them were aware of the difference compared to routine samples.

### 3.2. Management of Deviating Results during Routine Processing

The second part of the survey questioned laboratories’ common practices for handling problems in a routine setting (Table 3).

Similar to CAPA follow-up, mainly the pathologist and molecular biologist were involved in the interpretation of the test result and reporting of the observed findings. For 26.0% of responses (*n* = 435), the laboratories did not organize specific training for the persons performing the interpretation as required by ISO 15189 [3], besides their official educational degrees (Table 3). For the other cases, the most reported types of training included attending workshops or training courses (46.4%), performing validations (39.1%), or being trained by colleagues to carry out a certain procedure (35.2%). In the case of a test failure, the greater portion of institutes indicated that they always request a new sample from the requesting physician, but 31.4% (*n* = 388) of laboratories would not do this during the EQA scheme.

After the analysis, 75% (*n* = 300) of laboratories correlated their routine test results to relevant literature and 45.3% stored the results in their local pathology database (*n* = 289). The patient’s response to therapy was monitored with the treating physician for 39.7% (*n* = 302) of cases. Overall, 69.8% (*n* = 324) of laboratories organized some form of continuous education in the laboratory.

### 3.3. CAPA of Deviating Biomarker Results Related to Laboratory Characteristics

**Figure 1:**
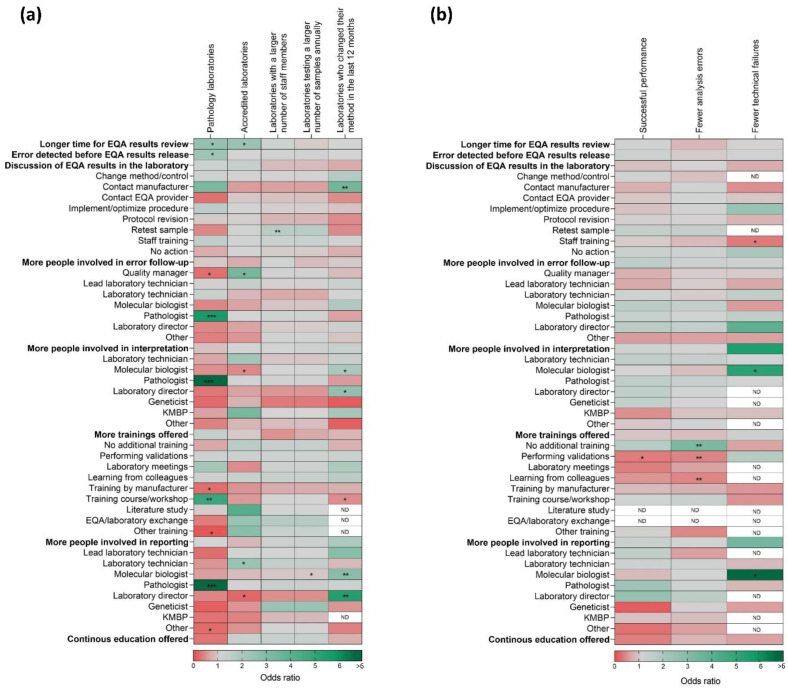
Survey answers related to probability of (**a**) different laboratory characteristics and (**b**) performance in the next EQA scheme; logistic regression models or proportional odds ratios with generalized estimating equations (GEE) were used. Odds ratios > (<) 1 reflect a higher (lower) probability for the action to be related to the laboratory characteristic/EQA performance. * *p* < 0.05, ** *p* < 0.01, *** *p* < 0.001. The number of laboratory characteristics and performance in the next EQA scheme used for calculation of the odds ratios is given in Table S4. Geneticists included cytogeneticists, medical geneticists, or clinical geneticists, as reported by the participants. Other responsible persons included (clinical/cell) biologists, engineers, bio-informaticians, molecular oncologists, product specialists, scientists, or team leaders (for technology platforms). Other training included foreign animal disease training, fine-tuning between personnel, or automated algorithms. (**a**) Laboratories under the department of pathology are those performing pathology reviews and the analytical tests in the same department, without outsourcing (a part of) the molecular test. Accreditation is defined as compliant to ISO 15189 or relevant national standards, such as CAP 15189 [2,3]. The action ‘additional participation’ (row level) is not shown as there were too few observations to perform statistical analysis. The link between the laboratory settings (university hospital, general hospital, etc.) and methodology used are not shown as they are categorical variables, and pairwise comparisons are described in the main text. (**b**) Successful performance was defined as a maximum of one analysis error in the total of analyzed samples (i.e., an analysis score of at least 90% for 10 samples or 88.8% for 9 samples). EQA, external quality assessment; KMBP, clinical molecular biologist in pathology (separate function in the Netherlands); ND, not determined because of too few observations.

In Figure 1, Panel a, we provide an overview of the probability (expressed in ORs) of adhering to a certain type of error management strategy mentioned in Table 2 and 3, depending on the survey respondents’ characteristics.

Laboratories performing all analyses only under the department of pathology (without outsourcing a part of the molecular test to another hospital or department, e.g., to a specialized genomics core) more frequently included a pathologist for interpretation, reporting, and management of deviating results. In addition, these laboratories had a higher probability of noticing the errors only after the EQA results were released, instead of in advance using an internal quality check. Both pathology laboratories and accredited laboratories took more time to evaluate the EQA results and to discuss them with colleagues in the laboratory after release by the EQA provider. Accredited laboratories included the quality manager in CAPA management.

Laboratories with a larger staff number more often retested a sample in case of deviating EQA results. Testing more samples annually increased the chance to involve a molecular biologist for reporting of the different test results.

Respondents who changed their testing method in the last 12 months prior to the survey were significantly more likely to obtain a deviating EQA result caused by that new methodology and to contact the respective manufacturer. In addition, they more frequently involved a molecular biologist and laboratory director in both the interpretation and reporting of the test results compared to laboratories who had not changed anything with their methodology in the past year (Figure 1, Panel a).

In this study, industry laboratories were more likely to have manufacturer-based training (*p* = 0.0257) and included fewer staff members in the interpretation of the results compared to clinical laboratories active in routine testing. They especially involved a pathologist less often for the interpretation of the result (*p* = 0.0443). Users of a commercial test methodology (based on next-generation sequencing (NGS), real-time polymerase chain reaction (PCR), FISH, or IHC) as expected were more likely to contact the manufacturer compared to users of LDTs (*p* = 0.0038, 12.249 [2.268; 66.160]) and were also less likely to revise their protocol (*p* = 0.0021, 0.365 [0.193; 0.691]). Users of NGS panels were more likely to involve a molecular biologist in the interpretation, reporting, and follow-up of problems (all *p* < 0.01) and more frequently trained their personnel by performing internal validations prior to testing (*p* < 0.005).

### 3.4. Improvement of Correct Testing in Next EQA Schemes

The probability of obtaining a successful performance or encountering an analysis error or test failure in the next EQA scheme depending on the practices for error management is displayed in Figure 1, Panel b. Laboratories in which a molecular biologist was involved in the interpretation (*p* = 0.0282, 0.194 [0.045; 0.836]) and reporting (*p* = 0.0321, 0.138 [0.023; 0.841]) of the obtained test result, obtained significantly less test failures in the next EQA scheme. In contrast, staff training performed as a CAPA to resolve the errors resulted in more test failures (*p* = 0.0409, 2.889 [1.046; 7.980]).

Respondents who did not offer specific training to people performing the interpretation in routine practice had a lower risk of obtaining an analysis error (*p* = 0.007, 10.309 [0.133;0.720]), while those being educated in the laboratory by other colleagues for a specific technique obtained more analysis errors (*p* = 0.0049, 2.286 [1.294; 4.037]). The introduction of new methods and performing of validations resulted in more analysis errors in the next scheme (*p* = 0.0014, 2.387 [1.414; 4.029]) and a lower chance of obtaining of a successful state (*p* = 0.0298, 0.365 [0.147; 0.904]) (Figure 1, Panel b). Laboratories performing in-depth validations more frequently used a complex NGS-based method (16.5%, *n* = 170) compared to laboratories who did not mention validation as part of the personnel training (1.9%, *n* = 256) (data not shown).

There was no significant relationship between any of the other elements and the performance criteria in the next scheme. In addition, while some elements influenced the probability to participate successfully or obtain fewer analysis errors or test failures, the average analysis score of the participants did not change significantly. The laboratory characteristics and performance in the next EQA scheme used for correlation with the strategies for error management are given in Table S4. The majority of survey respondents consisted of university and/or research laboratories (43.0%) affiliated to the department of pathology (84.0%). There was an almost equal distribution of accredited versus non-accredited laboratories (42.8%).

## 4. Discussion

The ISO 15189 standard specifies that laboratories should participate in interlaboratory comparisons and should perform appropriate CAPAs when predetermined performance criteria are not met [3]. Several groups have proposed the use of a well-structured framework for handling different types of deviating results [18,19].

### 4.1. Management of Deviating EQA Scheme Results

Our data demonstrated that the majority of respondents adhered to the elements specified in ISO 15189 to correct and prevent deviations compared to the validated EQA results (Table 2). In addition, CAPA management was not limited to accredited laboratories, even though the latter were more likely to include a quality manager in a review of results. Namely, for the 353 known and documented CAPAs, 149 (42.2%) were performed in accredited institutes, 163 (46.2%) in non-accredited institutes, and for 41 (11.6%) the accreditation status could not be determined. The fact that the molecular biologist and pathologist were the main designated staff members for follow-up can be explained as in most laboratories they are the end responsible persons for interpretation and reporting of the results, as confirmed by our data on management of routine biomarker tests (Table 3).

Our data also revealed that laboratories reported that they review the results actively, within a suitable timeframe and following discussion with other involved laboratory professionals. The variety of undertaken actions also depended on the cause reported for that specific error (Table S3). A detailed investigation of the root causes is described elsewhere [28]. While some causes such as clerical errors when completing the EQA datasheet seem accidental instead of systematic, the occurrence of such mistakes in routine practice could have detrimental effects on patient care. In this study, clerical errors were managed in 56.5% (26/46) of cases by implementing a second review step by another staff member to check the results before submitting them to the EQA provider.

Surprisingly, for 27.6% (142/514) of the cases, the laboratories reported that they do not undertake any CAPA. This did not depend on country, setting, or accreditation status, but occurred mainly in the case of a problem with the material (26.7%). The fact that 13% of cases were denoted as insufficient materials is surprising, as the materials were carefully validated beforehand and verified to contain sufficient neoplastic cells lacking tumor heterogeneity, and other peers were able to successfully analyze these cases in the same scheme. Indeed, pre-analytical variables such as the estimation of the neoplastic cell content has previously been linked to deviations in mCRC EQA results [29]. As the samples in the EQA scheme consisted of pre-cut and labelled samples, laboratories should therefore be extra cautious as other pre-analytical issues could arise in routine practice during sample reception, storage, and preparation.

Because of their pre-prepared nature, laboratories reported being aware that they were treating EQA samples. Nevertheless, the majority of respondents treated the samples identically compared to routine cases. It is only in this way that EQA schemes allow systematic shortcomings to be detected in the procedure by the EQA scheme that cannot be picked up by a laboratory’s internal quality check. The majority of participants therefore rated EQA participation to be important. However, as this survey was only sent to participants of EQA schemes this score might be lower for laboratories who refrained from participation, and it remains to be investigated how non-responders manage deviating test results.

It has been suggested that the feedback given by EQA providers to the participants is highly variable [19] and providers should include a checklist to improve their EQA schemes and aid laboratories in managing deviating results [18]. The ESP EQA schemes provide participants with detailed individual feedback, as well as a general report to allow them to compare to international peers and to the used test methods. In addition, educational good examples are provided for IHC stains and written reports. As proposed in the checklist/flowchart for error management [18,19], laboratories should identify if the root cause was internal to the laboratory or external, e.g., due to a mistake made by the EQA provider. Therefore, participants to the ESP schemes received one month time after release of the results to appeal their scores, after which scores were re-investigated, adapted if necessary, and an individually tailored response was formulated by the scheme experts.

### 4.2. Management of Deviating Results during Routine Processing

The second part of the survey also evaluated the general adherence to ISO 15189 for the management of test results in routine practice (not related to the specific error in any of the provided EQA samples) (Table 3). Even though the number of laboratories who offered training to the person performing the interpretation of routine test results was high, not all laboratories offered continuous education programs or performed dedicated training as required by the standard. Continuous education is especially important in predictive oncology, with the increasing complexity of new therapies and (panels of required) predictive biomarkers that are emerging, as well as frequent updates in recommendations [30]. Several studies have indeed highlighted an increased EQA performance for laboratories actively participating in workshops or group discussions compared to non-participants [31,32,33], as well as a positive effect of team meetings on oncology test outcomes [34]. In our study, there was no significant difference in trainings offered for accredited or non-accredited participants, however, pathology laboratories more frequently attended workshops compared to genetic laboratories (Figure 1, Panel a). In a routine setting, laboratories using complex techniques did perform more frequent validations. The increasing number of biomarkers to be tested and increase in NGS users indeed adds challenges for the interpretation of complex data [35,36]. None of the laboratories reported including a bio-informatician for EQA result interpretation, reporting, or review, in spite of 47/514 deviating results being obtained by NGS-based methods. This increasing complexity will further enhance the importance of involving experienced, well-trained experts (such as clinical scientists in molecular pathology) and discussing uncommon or rare test results at multidisciplinary meetings such as a molecular tumor board [37].

Our findings indeed reveal that the majority of laboratories stored their results in their own pathology database (Table 3), while 19.5% also attended such multidisciplinary meetings. In addition, the recorded test results were frequently (75.7%) correlated with findings in literature to evaluate the mutation rates as a quality indicator.

### 4.3. CAPA of Deviating Biomarker Results Related to Laboratory Characteristics

Besides the overall description of managing deviating EQA results, we also evaluated if the strategies differed depending on various routine laboratory characteristics (Figure 1, Panel a).

Even though previous findings reported an improved EQA performance for accredited institutes and institutes with a research setting [20], we did not observe a significant difference for both characteristics concerning EQA result review. The previously described positive effect of accreditation might therefore be related to the difference in root causes of the deviating results [28] and the fact that accredited laboratories are more likely to participate in quality improvement projects, as previously described [31]. As accredited laboratories took more time to review the results, it suggests that they are performing a more detailed analysis of root causes or that they discuss the results at a later moment during a dedicated quality meeting. However, more research is needed to find out the exact underlying causes of the previously reported better performance for accredited institutes. The fact that industry laboratories were more likely to attend manufacturer-based training and included fewer staff members in the interpretation of the results is not surprising, given that they are involved in the manufacturing of an assay instead of clinical diagnostic testing and they participate to the EQA program to evaluate their test’s performance.

Even though previous studies did not detect a difference in EQA performance for the different methods [20], we observed an effect on two levels. First of all, respondents who had changed their testing method in the last 12 months prior to the survey, more frequently contacted the respective manufacturer to resolve the problem. In addition, they more frequently involved a molecular biologist and laboratory director. These findings together with previously reported problems in the case of a recent method update, suggest that a change of test methodology is a profound problem that requires the input from expert laboratory professionals compared to more random errors.

Secondly, the type of methodology influenced the laboratory’s strategy to manage deviating EQA results, as users of a commercial test methodology (either for NGS, real-time PCR, FISH, or IHC) were more likely to contact the manufacturer compared to users of laboratory-developed tests (LDTs) and were less likely to revise their protocol. This was expected as these laboratories apply an automated setting and software provided in the manufacturer’s kit. This finding thus indicates that the presented data are reliable. Laboratories who tested a larger sample volume annually also more frequently retested the samples to confirm their submitted results, suggesting that they might have more resources for retesting the samples [38].

### 4.4. Improvement of Correct Testing in Next EQA Schemes

To assess if certain CAPAs resulted in an improvement of performance in the next EQA scheme, we evaluated the effect of CAPA management as reported by the participants on the state of successful performance, the number of analysis errors, and the number of test failures (Figure 1, Panel b). There was no negative impact of any of the CAPAs on these performance parameters. The involvement of a molecular biologist in routine result interpretation and reporting resulted in fewer test failures. Nevertheless, it must be noted that the overall number of test failures in the next EQA scheme was low (Table S4), which is why the ORs could not be computed for all the different elements.

Surprisingly, training of laboratory personnel (in the form of a CAPA, general training for interpretation of test results, or by continuous education) had a negative impact on the future scheme performance. This can be explained as these laboratories had more personnel-related errors, which were previously reported to be an influencing factor for diminished future EQA performance [28]. In addition, they used more complex testing techniques (such as NGS), which were shown to be more prone to method-based errors [39], as described in another paper focusing on the detailed error causes [28]. Laboratories should therefore not only focus on resolving specific errors, but implement preventive actions to improve the quality in the complete test process. Finally, we acknowledge that we included only those participants with at least one error in the EQA schemes. Therefore, our results might reflect the best practices in error management, as non-respondents might be less involved in quality improvement. Nevertheless, with data from 185 laboratories worldwide on 325 surveys and on 44.0% of cases with a deviating EQA result, we feel this is valuable first assessment of laboratories’ strategies in reviewing the EQA results, in light of the previously proposed frameworks [18,19]. Even though this is a study on EQA results for which pre-validated and prepared samples were sent, laboratories should investigate root causes and perform CAPAs for routine samples as well. It might thus be useful to further investigate quality management in routine institutes to verify if the variety in sample processing and types further enhances the variety of quality measures taken.

### 4.5. Recommendations for Future Error Management

This paper stressed the importance for laboratories to review the results actively, within a suitable timeframe and following discussion with other involved laboratory professionals. As the adherence to the ISO 15189 standard for error follow-up was high, but not limited to accredited institutes, there is currently insufficient evidence to state that accreditation resulted in better error management. However, based on the longer time for EQA result review and more in-depth discussion with laboratory personnel, it is advised that EQA findings are thoroughly discussed in the laboratory as this should be performed in a routine setting. Most importantly, there are still a number of institutes not performing any CAPA (27.6% of cases), especially in the case of pre-analytic issues. Even in the case of such pre-analytical issues, laboratories are recommended to actively use the EQA feedback and resources to further investigate the root cause of these issues, as even more challenging samples might occur in a routine setting. The importance of these early steps was demonstrated by the fact that pathology laboratories (who also had more material-related issues) had a higher probability of detecting the problem too late.

The fact that these sample-related issues as well as issues arising from (changing to) complex techniques were resolved by including dedicated personnel, stresses the importance of communication between stakeholders within the laboratory, such as pathologists, molecular biologists, laboratory directors to determine the optimal test strategy, and everyone involved in follow-up of the test results. With the increasing complexity of predictive testing in clinical oncology, the appointment of an expert molecular biologist (e.g., clinical scientist in molecular pathology) as a permanent member of the pathology team responsible for (molecular) predictive testing is essential [40]. In-depth validations should be performed for complex or LDT methods. In the case of commercial methods, laboratories should contact the manufacturer to get to the root cause of the error when the solution is beyond the laboratories’ capabilities. In the case of doubt, laboratories can retest a sample to prevent deviating results.

The use of EQA schemes as a tool to reduce random errors (such as clerical mistakes) was also demonstrated by the high fraction of laboratories implementing a second review step to prevent this occurrence in the future. Even when no incidents occur related to the test methodology or equipment, training of laboratory personnel remains the first step to achieving consistently accurate test results in order to prevent deviating results downstream of the test process.

## 5. Conclusions

This is the first time that a longitudinal evaluation of CAPA questionnaires has been performed and different error management strategies were identified that would otherwise not have been detected by merely checking the scheme’s error rates. The surveys were shown to be feasible for EQA providers to request more information on CAPAs by laboratories for further follow-up of EQA results, for instance in the case of poor performance, and demonstrated a wide variety in actions performed for follow-up. It remains to be elucidated how non-responding laboratories handle deviating test results from routine and EQA samples. Based on these findings, we formulated some recommendations for laboratories to follow up on deviating EQA results and application in a routine setting, to ultimately improve test practices.

## Figures and Tables

**Table 1:** Number of cases analyzed per subscheme offered in the European Society of Pathology (ESP) external quality assessment (EQA) schemes.

Year			2015	2016	2017	2018	Study Total
Analyzed laboratories	EQA participations to different subschemes		329	445	733	712	2219
	Unique laboratories participating		197	234	259	241	410 *
	Unique laboratories who received the survey (laboratories with at least one error in any of the subschemes)		88	120	153	175	315 *
	Unique laboratories who replied to the survey		39	44	90	99	185 *
Analyzed surveys	Surveys sent		105	154	234	298	791
	Surveys with response		40	53	108	124	325
Analyzed cases	Cases tested in the scheme		4224	5134	6276	5902	21536
	Deviating EQA results included in survey		162	225	362	418	1167
	Deviating EQA results with response		51	74	181	208	514
NSCLC	FISH	*ALK*	2	4	18	1	25
		*ROS1*	6	3	7	33	49
	FISH digital	*ALK*	1	0	4	2	7
		*ROS1*	4	3	5	21	33
	IHC	ALK	0	4	20	7	31
		ROS1	9	0	2	6	17
		PD-L1	N/A	N/A	35	28	63
	IHC technical *	ALK	8	6	7	15	36
		ROS1	N/A	1	0	2	3
		PD-L1	N/A	N/A	N/A	6	6
	IHC digital	PD-L1	N/A	N/A	11	19	30
	Variant analysis	*EGFR* (mandatory)	21	19	41	27	108
		*KRAS* (optional)	N/A	N/A	2	11	13
		*BRAF* (optional)	N/A	N/A	0	3	3
mCRC	Variant analysis	*KRAS* (mandatory)	N/A	23	23	22	68
		*NRAS* (mandatory)	N/A	7	2	3	12
		*BRAF* (optional)	N/A	4	4	2	10

Laboratories were free to participate in one of the techniques for a selected marker. Participation to FISH digital was mandatory for the same marker if a laboratory registered for FISH for that marker, and participation to IHC digital or technical was mandatory for IHC participants for the same marker. * One unique laboratory could have participated, received the survey, and replied to the survey in several scheme years, which is why the total number of unique participants does not equal the sum of the different years. N/A: not applicable as no surveys were sent (no EQA scheme offered or only a pilot or scheme outside the study period). *ALK*, ALK receptor tyrosine kinase; *BRAF*, B-Raf proto-oncogene; *EGFR*, epidermal growth factor receptor; EQA, external quality assessment; ESP, European Society of Pathology; FISH, fluorescence in-situ hybridization; IHC, Immunohistochemistry; *KRAS*, KRAS proto-oncogene; mCRC, metastatic colorectal cancer; *NRAS*, NRAS proto-oncogene; NSCLC, non-small cell lung cancer; PD-L1, programmed death ligand 1; *ROS1*, ROS proto-oncogene 1.

**Table 2:** Laboratories’ practices in processing feedback on cases with deviating EQA results, related to ISO 15189 requirements.

Question	*n* (%)	ISO 15189:2012 Clause [3]
**When are EQA results evaluated (time after release by the provider)?**	**407**	**5.6.3.1**
Within a week	238 (58.5)	
Within two weeks‒within a month	163 (40.0)	
More than 1 month	6 (1.5)	
**When was the error detected?**	**435**	**5.6.2.3** **5.7.1**
Before release of the EQA report	70 (16.1)	
After release of the EQA report	365 (83.9)	
**Are the results discussed in the laboratory?**	**330**	**4.1.2.6** **5.6.3.4**
Yes, always	270 (81.8)	
Only in the case of deviating results	54 (16.4)	
No	6 (1.8)	
**Which action was performed for this specific error?**	**514**	**5.6.3.4**
Additional EQA participation	7 (1.4)	
Change method/control tissue	48 (9.3)	
Contact manufacturer	38 (7.4)	
Contact EQA provider	17 (3.3)	
Implement/optimise procedure	64 (12.5)	
Protocol revision	75 (14.6)	
Retest sample	26 (5.1)	
Staff training	78 (15.2)	
Unknown	19 (3.7)	
None	142 (27.6)	
**How many people were involved in the follow-up of the deviating result for this case?**	**430**	**4.1.2.5**
1	241 (56.0)	
2	114 (26.5)	
3 or more	75 (17.4)	
**Who was responsible for this action? ^a^**	**430**	**4.9**
Quality manager	50 (11.6)	
Lead laboratory technician	72 (16.7)	
Laboratory technician	96 (22.3)	
Molecular biologist	172 (40.0)	
Pathologist	200 (46.5)	
Laboratory director	87 (20.2)	
Other	32 (7.4)	
**Were the EQA samples treated differently in any way?**	**315**	**5.6.3.3**
No	265 (84.1)	
Yes	50 (15.9)	
**Was the personnel aware they were handling EQA samples?**	**318**	**5.6.3.3**
No	28 (8.8)	
Yes	290 (91.2)	
**Importance given to EQA participation**	**318**	**4.2.2** **5.6.4**
7 or less	31 (9.7)	
8	49 (15.4)	
9	51 (16.0)	
10	187 (58.8)	

^a^ Percentages add up to more than 100% as more than one type of training could be reported. An explanation for the different relevant clauses is presented in Table S2. Other responsible persons included (clinical/cell) biologists, engineers, bio-informaticians, molecular oncologist, product specialist, scientist, or a team leader (technology platforms). EQA, external quality assessment; ISO, International Organization for Standardization; *n*, number.

**Table 3:** Laboratories’ practices for handling of test results in a routine setting, irrespective of errors in the EQA scheme.

Question	*n* (%)	ISO 15189:2012 Clause [3]
**Who interprets the results? ^a^**	**474**	**4.1.2.1**
Lead laboratory technician	21 (4.4)	
Laboratory technician	83 (17.5)	
Molecular biologist	215 (45.4)	
Pathologist	308 (65.0)	
Laboratory director	40 (8.4)	
KMBP/molecular pathologist	19 (4.0)	
Geneticist	13 (2.7)	
Other	23 (4.9)	
**Is additional training received to perform the interpretation? ^a^**	**435**	**5.1.6** **5.1.9**
No additional training, degree only	113 (26.0)	
Performing validations (internal)	170 (39.1)	
Laboratory meetings (internal)	98 (22.5)	
Learning from colleagues with gradually more independence (internal)	153 (35.2)	
Training by the manufacturer (external)	33 (7.6)	
Training course/workshop (external)	202 (46.4)	
Literature study (internal)	9 (2.1)	
EQA/laboratory exchange (internal)	10 (2.3)	
Other training	6 (1.4)	
**Who reports the results? ^a^**	**460**	**5.8.1** **5.9.1**
Lead laboratory technician	13 (2.8)	
Laboratory technician	62 (13.5)	
Molecular biologist	159 (34.6)	
Pathologist	314 (68.3)	
Laboratory director	41 (8.9)	
KMBP/molecular pathologist	8 (1.7)	
Other	31 (6.7)	
**Is an additional sample always requested?**	**388**	**4.7**
No	45 (11.6)	
Depends on sample availability/type	9 (2.3)	
Yes in routine practice but not for EQA	122 (31.4)	
Yes, always	212 (54.6)	
**Do you submit your results to a database?**	**289**	**5.7.1**
No, never	15 (5.2)	
No, a report for the oncologist is made only	95 (32.9)	
No, our results are research use only	7 (2.4)	
Yes, unspecified	9 (3.1)	
Yes, a local oncology database with patient follow-up	7 (2.4)	
Yes, a national pathology database	25 (8.7)	
Yes, our local pathology database	131 (45.3)	
**Do you correlate molecular results with relevant literature?**	**300**	**4.1.2.2**
No	73 (24.3)	
Yes	227 (75.7)	
**Do you ask for follow-up of the patient’s results?**	**302**	**4.14.7**
No	77 (25.5)	
No, although I would be interested	105 (34.8)	
Yes, unspecified	14 (4.6)	
Yes, during a multi-disciplinary team meeting	59 (19.5)	
Yes, occasionally for patients with specific variants	47 (15.6)	
**Do you take part in continuous education?**	**324**	**5.1.8**
No	98 (30.2)	
Yes	226 (69.8)	

^a^ Percentages add up to more than 100% as more than one type of training could be reported. An explanation for the different relevant clauses is presented in Table S2. Geneticists included the terms cytogeneticist, medical geneticist, or clinical geneticist as reported by the participants. Other responsible persons included (clinical/cell) biologists, engineers, bio-informaticians, molecular oncologist, product specialist, scientist, or a team leader (technology platforms). Other training includes foreign animal disease training, fine-tuning between personnel, or automated algorithms. EQA, external quality assessment; ISO, International organization for standardization; KMBP, clinical molecular biologist in pathology (separate function in the Netherlands); *n*, number.

## Supplementary Materials

The following are available online at https://www.mdpi.com/2075-4418/10/10/837/s1, Table S1: Overview of survey questions, Table S2: Relevant clauses in ISO 15189 included in Table 2 and 3, Table S3: Overview of actions undertaken depending on the cause of the deviating EQA result, Table S4: Laboratory characteristics and scores in next EQA scheme for the study cases.
