# Supplementary material for: Managing Deviating EQA Results: A Survey to Assess the Corrective and Preventive Actions of Medical Laboratories Testing for Oncological Biomarkers
Source: Diagnostics (Basel). 2020 Oct 18;10(10):837. doi: 10.3390/diagnostics10100837 (PMC7603102; doi:10.3390/diagnostics10100837)
Supplement: Supplementary file 1 [file diagnostics-10-00837-s001.pdf]

Supplementary Materials

Table S1. Overview of survey questions.

General questions 2–4 were initially included in the survey, but in later schemes they were included in the EQA datasheets and linked to the survey answers for the identical participants. *ALK*, ALK receptor tyrosine kinase; EQA, external quality assessment; FISH, fluorescence in-situ hybdrisation; SOP, standard operating procedure; Q, question.

INTRODUCTION: Please find below a short questionnaire on the follow-up the 2018 Lung EQA scheme results. You can select the appropriate option in the dropdown menu, which will become visible when clicking on 'please choose'. If required, please specify your answer in more detail in the column next to the selected option. Your contribution to this study is highly appreciated.
EQA ID: *Prefilled with participants anonymous number to the EQA scheme* e.g., *2018LUNG0001*

- o One answer possible
- Multiple answers possible

A. <u>CASE-SPECIFIC QUESTIONS</u> *Repeated for all cases in which an error occurred*

SUBSCHEME: *Prefilled with relevant marker and subscheme* e.g., *ALKFISH*
CASE NR: *Prefilled with the sample label and error type* e.g., *L18.ALKFISH1: false-negative*

Q1: During which phase in the test process did the error occur?

- o Pre-analytical phase
- o Analytical phase
- o Post-analytical phase
- o I don't know

Q2: What was the cause of this error?

- o Clerical error
- o Methodological problem
- o Equipment problem
- o Technical problem
- o Reagent problem
- o Personnel error
- o Interpretation error
- o Problem with EQA material
- o I don't know because not documented
- o I don't know although documented
- o Other (please specify)

Q3: Please shortly specify the cause of the error:

Q4: Which corrective/preventive action was taken for this error?

- o Implement/optimize documentation (procedure)
- o Protocol revision (technical)
- o Change method/control tissue
- o Contact company
- o Staff training (internal)
- o Staff training (external)
- o None
- o I don't know
- o Other (please specify)

Q4.1: If staff training, please clarify which kind:
e.g., Additional EQA participation, workshop, internal SOP revision,..

Q5: Who was responsible for this action?

Quality manager
Lead laboratory technician
Laboratory technician
Molecular biologist
Pathologist
Laboratory director
Bio-informatician
Other (please specify)

Q6: Was this error detected before or after the EQA results were released?

- o Before
- o After

Q6.1: If before, please specify how the error was identified:

Q7: Do you think about changing your test method in the next year?

- o Yes
- o No

Q7.1: If yes, please specify the new method:

Q8: Comments (optional):

B. <u>GENERAL QUESTIONS</u> *To be completed only once*

Q1: Did you change anything to the test protocol/method in the last 12 months?

- o Yes
- o No

Q1.1: If yes, please specify what was changed:

Q2: Who interprets the results?

Quality manager
Lead laboratory technician
Laboratory technician
Molecular biologist
Pathologist
Laboratory director
Bio-informatician
Other (please specify)

Q3: How is this person trained to interpret the results?

Internal: performing validations
Internal: learning from colleagues with gradually more independence
Internal: participate to laboratory meetings
Internal: participate to laboratory meetings
External: workshop
No additional training, but learned from degree
Other (please specify)

Q4: Who reports the results?

Quality manager
Lead laboratory technician
Laboratory technician
Molecular biologist
Pathologist
Laboratory director
Bio-informatician
Other (please specify)

Q5: On average, how long after release of the EQA report are the results reviewed?

- Within a week after the release of the EQA report
- Within a month after release of the EQA report
- 1 month after release of the EQA report
- Only when there is an upcoming external audit
- Other (please specify)

Q6: Are the EQA results discussed with other people from the laboratory in a meeting?

- Yes, always
- Only in case of problematic results
- No
- Other (please specify)

Q7: Do you organize continuous education related to quality assurance in the lab?

- Yes
- No

Q7.1: If yes, please specify:

Q8: On a scale from 1 to 10 (10 being the most important) how important do you rate participation to EQA?

- 1
- 2
- 3
- 4
- 5
- 6
- 7
- 8
- 9
- 10

Q9: Are the EQA samples treated differently in any way compared to routine diagnostic samples?

- Yes
- No

Q9.1: If yes, please specify how:

Q10: Are the persons working in the laboratory aware that they are handling samples for EQA?

- Yes
- No

Q11: Do you request an additional sample in case a technical failure occurred?

- o Yes, always
- o Yes in routine practice but not for EQA
- o No

Q12: Do you correlate the molecular results with the clinical context (e.g., Frequency of variants)?

- o Yes
- o No

Q13: Do you ask for follow-up results of the patient with response to therapy?

- o Yes, occasionally for patients with specific variants
- o Yes, during a multi-disciplinary team meeting.
- o No
- o No, although I would be interested.
- o Other (please specify)

Q14: Do you submit your results to a database?

- o No, a report for the oncologist is made only.
- o Yes, our local pathology database.
- o Yes, a national pathology database.
- o Yes, a local oncology database with patient follow-up
- o Other (please specify)

THANK YOU FOR YOUR PARTICIPATION.

Table S2. Relevant clauses in ISO 15189 included in Table 2 and 3 [3].

ISO 15189 Clause	Description
4.1.2.1	Laboratory management shall continually improve its effectiveness by ensuring that all personnel are competent to perform their assigned activities and shall document personnel qualifications for each position. The qualifications shall reflect the appropriate education, training, experience and demonstrated skills needed, and be appropriate to the tasks performed.
4.1.2.2	Laboratory management shall ensure that laboratory services, including appropriate advisory and interpretative services, meet the needs of patients and those using the laboratory services.
4.1.2.5	Laboratory management shall ensure that responsibilities, authorities and interrelationships are defined, documented and communicated within the laboratory organization. This shall include the appointment of person(s) responsible for each laboratory function and appointment of deputies for key managerial and technical personnel. NOTE: in smaller laboratories individuals can have more than one function and that it could be impractical to appoint deputies for every function.
4.1.2.6	Laboratory management shall have an effective means for communicating with staff, records shall be kept of items discussed in communications and meetings.
4.2.2	The quality management system shall include, but not be limited to, internal quality control and participation in organized interlaboratory comparisons such as external quality assessment schemes.
4.7	The laboratory shall establish arrangements for communicating with users on consulting on scientific and logistic matters such as instances of failure of sample(s) to meet acceptance criteria.
4.9	The laboratory shall have a documented procedure to identify and manage nonconformities. The procedure shall ensure that the responsibilities and authorities for

	handling nonconformities are designated, and that the immediate actions to be taken are defined.
4.14.7	The laboratory should establish quality indicators for systematically monitoring and evaluating the laboratory's contribution to patient care.
5.1.6	Following appropriate training, the laboratory shall assess the competence of each person to perform assigned managerial or technical tasks according to established criteria. Reassessment shall take place at regular intervals. Retraining shall occur when necessary.
5.1.8	A continuing education programme shall be available to personnel who participate in managerial and technical processes. Personnel shall take part in continuing education. The effectiveness of the continuing education programme shall be periodically reviewed. Personnel shall take part in regular professional development or other professional liaison activities.
5.1.9	Records of the relevant educational and professional qualifications, training and experience, and assessments of competence of all personnel shall be maintained.
5.6.2.3	The laboratory shall have a procedure to prevent the release of patient results in the event of quality control failure.
5.6.3.1	The laboratory shall monitor the results of the interlaboratory comparison programme(s) and participate in the implementation of corrective actions when predetermined performance criteria are not fulfilled.
5.6.3.3	The laboratory shall integrate interlaboratory comparison samples into the routine workflow in a manner that follows, as much as possible, the handling of patient samples. Interlaboratory comparison samples shall be examined by personnel who routinely examine patient samples using the same procedures as those used for patient samples.
5.6.3.4	When predetermined performance criteria during interlaboratory comparisons are not fulfilled (i.e., nonconformities are present), staff shall participate in the implementation and recording of corrective action. The effectiveness of corrective action shall be monitored. The returned results shall be evaluated for trends that indicate potential nonconformities and preventive action shall be taken. The performance in interlaboratory comparisons shall be reviewed and discussed with relevant staff.
5.6.4	The laboratory shall participate in interlaboratory comparisons such as those organized by external quality assessment schemes.
5.7.1	The laboratory shall have procedures to ensure that authorized personnel review the results of examinations before release and evaluate them against internal quality control and, as appropriate, available clinical information and previous examination results.
5.8.1	The results of each examination shall be reported accurately, clearly, unambiguously and in accordance with any specific instructions in the examination procedures.
5.9.1	The laboratory shall establish documented procedures for the release of examination results, including details of who may release results and to whom.

Table S3. Overview of actions undertaken depending on the cause of the deviating EQA result.

	**Cause of deviating EQA result**							
	**Interpretation Error (*n* = 144)**	**Methodological Problem (*n* = 105)**	**Problem with EQA Material (*n* = 67)**	**Reagent Problem (*n* = 52)**	**Clerical Error (*n* = 46)**	**Personnel Error (*n* = 36)**	**Technical Problem/ Equipment (*n* = 25)**	**Unknown/ Other (*n* = 39)**
Staff training (*n* = 78)	60.3	1.3	5.1	3.8	3.8	21.8	1.3	2.6
Protocol revision (*n* = 75)	14.7	18.7	10.7	28.0	1.3	12.0	10.7	4.0
Implement/ optimise procedure (*n* = 64)	26.6	7.8	7.8	1.6	40.6	9.4	1.6	4.7
Change method/ control (*n* = 48)	6.3	56.3	6.3	20.8	0.0	0.0	8.3	2.1
Retest sample (*n* = 26)	53.8	3.8	15.4	0.0	0.0	7.7	3.8	15.4
Contact manufacturer (*n* = 38)	18.4	44.7	0.0	21.1	0.0	0.0	10.5	5.3
Contact EQA provider (*n* = 17)	23.5	17.6	17.6	0.0	11.8	0.0	23.5	5.9
Additional EQA participation (*n* = 7)	42.9	0.0	14.3	14.3	28.6	0.0	0.0	0.0
Unknown (*n* = 19)	21.1	15.8	5.3	0.0	15.8	0.0	0.0	42.1
None (*n* = 142)	23.9	23.9	26.8	5.6	6.3	1.4	1.4	10.6

Abbreviations: EQA, external quality assessment.

Table S4. Laboratory characteristics and scores in next EQA scheme for the study cases.

Laboratory Characteristics	Number of Respondents/Cases	% of rEspondents/Cases (n = 514)
**Setting**		
Industry	20	3.9
(private) laboratories	122	23.7
Hospital laboratories	151	29.4
University and/or research	221	43.0
**All analyses under department of pathology**		
Yes	432	84.0
No	75	14.6
Missing data	7	1.4
**Accreditation**		
Accredited	243	47.3
Not accredited	220	42.8
Missing data	51	9.9
**Number of staff members involved in biomarker test**		
1–5	234	45.5
6–10	135	26.3
11–20	89	17.3
>20	23	4.5
Missing data	33	6.4
**Number of routine samples tested annually per biomarker**		
No clinical samples tested	12	2.3
<10	15	2.9
10–99	119	23.2
100–249	112	21.8
250–499	74	14.4
>500	33	6.4
Missing data	149	29.0
**Change in test method in the last 12 months**		
No	339	66.0
Yes	109	21.2
Missing data	66	12.8
**Methods of analysis**		
**Commercially available method**	257	50.0
ALK FISH	25	4.9
ROS1 FISH	48	9.3
ALK IHC	23	4.5
PDL1 IHC	35	6.8
Variant analysis	126	24.5
**Laboratory-developed method**	140	27.2
ALK IHC	44	8.6
PD-L1 IHC	34	6.6
ROS1 FISH	1	0.2
ROS1 IHC	20	3.9
Variant analysis	41	8.0
**Next-generation sequencing**	47	9.1
**Not analyzed because error in digital case**	70	13.6
**Successful performance in next EQA scheme**		
Yes	147	28.6
No	52	10.1
Not determined*	315	61.3
**Analysis error in next EQA scheme**		
Yes	63	12.3

No	149	29.0
Not determined*	302	58.8
**Test failure in next EQA scheme**		
Yes	16	3.1
No	196	38.1
Not determined*	302	58.8

*The performance criteria in the next EQA scheme could not be determined for all laboratories as not all laboratories registered in the next scheme. Abbreviations: *ALK*, ALK receptor tyrosine kinase; EQA, external quality assessment; FISH, fluorescence in-situ hybridization; IHC, Immunohistochemistry; PD-L1, programmed death ligand 1; ROS1, ROS proto-oncogene 1.
